# Complications of aortic coarctation repair assessed using cardiac magnetic resonance imaging

**DOI:** 10.1186/1532-429X-11-S1-P247

**Published:** 2009-01-28

**Authors:** Sylvia SM Chen, Raad H Mohiaddin

**Affiliations:** Royal Brompton Hospital, London, UK

**Keywords:** Cardiac Magnetic Resonance, Balloon Dilatation, Cardiac Magnetic Resonance Imaging, Aortic Coarctation, Residual Stenosis

## Introduction

Coarctation of the aorta (CoA) accounts for up to 10% of all congenital heart defects. Open repair with surgical reconstruction has been the main form of treatment and more recently, aortic stenting has been used as a less invasive technique without the need for cardiac bypass. The long term success and impact of repair on the structure of the aorta is important with regard to morbidity and management of these patients.

## Purpose

We used cardiac magnetic resonance imaging (CMR) to assess the long term success of repair of CoA with respect to structural complications and residual stenosis.

## Methods

CMR studies performed in patients with repaired CoA from January 2005 to September 2008 were reviewed. Details of age at, date and type of repair were obtained from patient medical notes. Multi-slice HASTE, cine and turbo-spine echo T2 images, and aortic in-plane and through-plane flow studies were analysed for complications of repair (aneurysmal dilatation, false aneurysm, dissection) and residual stenosis. Aneurysmal dilatation was defined as localised widening at the repair site in comparison to the diameter of the pre- and post-aortic segments. Residual stenosis was defined as constriction at the repair site with flow acceleration on in-plane flow analysis, ± peak recorded velocity on through-plane measurement of >1.5 m/s.

## Results

Analyses were done on 281 studies (167 males, 114 females, aged 31 ± 9 years). Average time between surgical repair and balloon dilatation, and CMR imaging 18 ± 7 years, and between aortic stenting and CMR imaging 4 ± 0.8 years. Types of repair: resection and end-to-end anastomosis (n = 94, 83%), subclavian flap repair (n = 62, 22%), aortic stenting (n = 43, 15%), interposition graft repair (n = 31, 11%: 22 dacron, 9 gelseal), balloon dilatation (n = 24, 9%), dacron patch repair (n = 19, 7%), and bypass graft (n = 8, 3%). Structural complications were most marked in patch repair (15/19 aneurysmal dilatation within the patch, 2/19 suture line false aneurysms, 2/19 residual stenosis), and subclavian flap repair (38/62 aneurysmal dilatation within the flap, 2/62 suture line false aneurysms, and 2/62 residual stenosis). There were no direct structural complications from resection and end-to-end anastomosis, but 57/94 had residual stenosis. Balloon dilatation had 17/24 residual stenosis and mild aneurysmal dilatation in 4/24. Aortic stenting showed less residual stenosis (6/43), with minimal structural complications (no dissection, and only 1/43 displacement of the stent, 1/43 aneurysm). Reasonably good long term results were observed in graft repairs: interposition grafts (3/31 suture line false aneurysms, 4/31 residual stenosis at arch and graft anastomosis) and bypass grafts (none with residual stenosis, and 2/8 suture line false aneurysm). Main complications of the types of repair are in Table 1.

**Table 1:** Types of aortic coarctation repair and the main complications

Repair	Aneurysmal dilatation (%within the type of repair)	Suture line false aneurysm (%within type of repair)	Residual stenosis (%within type of repair)
Dacron patch	79%	10%	10%
Subclavian flap	61%	3%	3%
Resection and end-to-end anastomosis	0%	0%	61%
Balloon dilatation	16%	N/A	70%
Aortic stenting	2%	N/A	13%
Interposition graft	0%	9%	13%
Bypass graft	0%	25%	0%

## Conclusion

Patch and flap aneurysms are frequent complications of patch and subclavian flap repair of aortic coarctation, and the rate of residual stenosis with resection and end-to-end anastomosis was high. Balloon dilatation had minimal complications, but frequent residual stenosis. Graft and bypass graft repair are the most favourable surgical repairs with limited complications and residual stenosis. Aortic stenting also appears quite successful with few complications and residual stenosis but long term follow-up is not yet available.

